# Mental Health Care and Mental Health Legislation in Pakistan: No Mercy for Losers

**DOI:** 10.1371/journal.pmed.0020397

**Published:** 2005-11-29

**Authors:** Haider Ali Naqvi

**Affiliations:** **1**Aga Khan University HospitalKarachiPakistan

In the Policy Forum by Gilani et al. [1], the authors have quite vividly described the state of mental health care and legislation in Pakistan. Indeed, the situation is one of low awareness and resources, much like other low-income countries in Southeast Asia. Even at the international level, there is deep-seated societal ambivalence toward the mentally ill. The so-called human rights principles have little material effect on the lives of psychiatric patients, and create double standards in the exercise of choice [2].

The promulgation of the new mental health ordinance has, indeed, been a red-letter day in the history of Pakistani legislation. However, how this document translates into real world action remains to be seen. In Pakistan, 70% of health-care services are provided by the private sector, and this, too, is mostly curative in nature [3]. According to the World Health Report (2004), 100% of health-care payments are an out-of-pocket expense for Pakistanis. Most private health care is unregulated. No hospital in Pakistan has Joint Commission on Accreditation on Healthcare Organization (JCAHO) accreditation. Anecdotal reports on abuse of individuals who are mentally ill are ubiquitous. It is not uncommon to see patients who are mentally ill chained to their beds. There is poor provision of psychotropic medication in government-run hospitals. Contrarily, one sees a cocktail of five medications prescribed by an inadequately trained mental health professional in private practice. Out of 342 registered psychiatrists, hardly 100–150 have adequate training. The Pakistan Medical and Dental Council is the sole body for the proper licensing and credentialing of physicians. The problem lies in the implementation of rules and regulations, rather than their existence. One sees a chain of psychiatric hospitals, claiming to deliver psychiatric care, with no qualified psychiatrists on their panels. There is no legal action taken against these people who blatantly exploit patients with mental illness.

All the major tertiary care centers in Pakistan have allied general medical and anesthesia services, yet the provision of modified electroconvulsive therapy (ECT) is deemed only “preferable” in the new mental health ordinance. Unmodified use of ECT results in serious and potentially life-threatening complications. Similarly, there are other paradoxes in the actual care and legislative protection of people with mental illness. The Federal Authority for Mental Health has played no active role in addressing these glaring inequities since its organization in 2001. Contrarily, there is a risk that it might become a power broker for bureaucracy and ministry officials, rather than serving the real stakeholders.

Essentially, nothing has changed for people with mental illness, except the nomenclature and terminologies. There is still no mercy for people with mental illness in poor and other marginalized communities.

Governmental low health-care spending (less than 1% of gross national product) should be seen in the context of the bigger geopolitical situation. Countries' major spending is on defense and military armaments. This is in a politically volatile environment, with ongoing border conflicts with neighbors. However, there is a need for strong political will from the government, which oversees the implementation of rules and regulations and protects the rights of people with mental illness.

For a comprehensive solution, an active public–private partnership is required. This requires a unified agenda and commitment from both sectors. Any lasting solution has to address the deep-rooted inequities, ethical misconduct, and micro- and macroeconomic issues.

## References

[pmed-0020397-b1] Gilani AI, Gilani UI, Kasi PM, Khan MM (2005). Psychiatric health laws in Pakistan: From lunacy to mental health. PLoS Med.

[pmed-0020397-b2] Harding TW (2000). Human rights law in the field of mental health: A critical review. Acta Psychiatr Scand.

[pmed-0020397-b3] Khattak FH (1996). Financing of health sector in health economics and planning in Pakistan.

